# Clear Cell Sarcoma: A Case Report and Review of Literature

**Published:** 2018-01-01

**Authors:** Alireza Abdollahi, Fatemeh Khatami, Seyed Mohammad Tavangar

**Affiliations:** 1 Department of Pathology, School of Medicine, Tehran University of Medical Sciences, Tehran, Iran; 2 Chronic Diseases Research Center, Endocrinology and Metabolism Population Sciences Institute, Tehran University of Medical Sciences, Tehran, Iran; 3 Department of Pathology, Shariati Hospital, School of Medicine, Tehran University of Medical Sciences, Tehran, Iran

**Keywords:** Clear cell sarcoma, Soft tissues, Malignant melanoma, Melanoma of soft part

## Abstract

Clear cell sarcoma (CCS), a deep-rooted tumor with a predilection for lower extremities, has a proclivity to involve the tendons and aponeuroses. This sarcoma is seen predominantly around the foot and ankle region. Diagnosis is mainly finalized using histological and immunohistochemical assessment. The main treatment strategy is surgery followed by chemotherapy. An erratic challenge is posed by histological similarity and immunohistochemical overlap to the diagnosis and distinguishing of clear cell sarcoma from primary or metastatic malignant melanoma (MM) which is more common. Here, we described a CCS case located in the left leg of a 37-year-old male patient.

## Introduction

 Clear cell sarcoma (CCS) is a rare neoplasm with a problematic clinical and histological distinction diagnosis. It is also known as malignant melanoma of soft part  representing about 1% of soft tissue tumors that classically involves tendons and aponeuroses with a prediction for the lower extremities^   1 ^. This sarcoma, can be seen particularly around the foot and ankle region^   1 ^. In fact, the major cause of this tumor remains unidentified; however, there are some investigations indicating that CCS could be the consequence of radiotherapy^   2 ^. CCS may occur at any ages, but a limited number of reports have been published regarding the incidence risk of CCS over the age of 40 years^3^^, ^^4^. This tumor is slightly more common in women than in men^   5 ^. The primary suspicion to the diagnosis of CCS can be achieved by imaging studies, but the exact diagnosis is mainly finalized using histological assessment^   5 ^. Similar to other invasive malignancies, CCS may quickly metastasize to other organs, especially to the lungs, so regular monitoring is crucial to follow-up tumor progression^5^.The original treatment plan for CCS is surgery followed by chemotherapy^   6 ^. However, several factors including position, grade and tumor size can finalize the treatment plan^   6 ^. After the treatment, the patients' following-up should be considered every three months up to two years^   6 ^. Herein, we present a case of CCS located in the left leg of a 37-year- old man. 

**Case Presentation**

**Figure 1:**
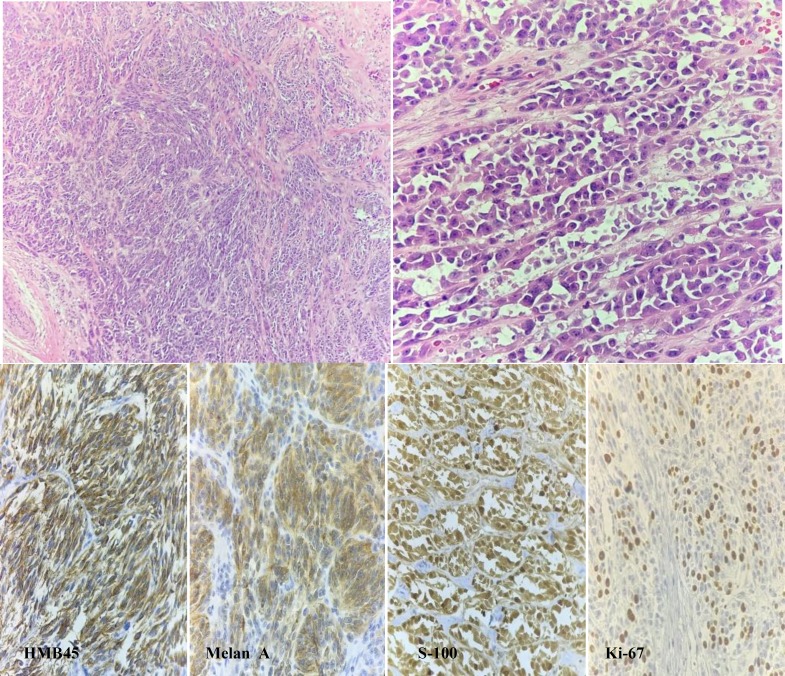
The microscopic study of the tumor consists of cellular nests which were separated by fibrous band from each other. The forms of cells vary from round-shaped to fusiform with vesicular nucleus, prominent nucleolus and granular eosinophilic cytoplasm, mitosis were seen. Lymphocytes cells are often seen on fibrosis bands. In immunohistochemical staining, antibodies that were in keeping with clear cell carcinoma were positive against the following markers: S-100, Ki-67, Melan A & HMB45.

Our case was a 37-year-old man without previous history of chronic disorders such as hypertension, diabetes mellitus, rheumatologic disorders or cardiovascular diseases. About one year ago, he realized a dull pain in his left leg followed by claudication. The initial physical examination raised doubts about the mass in his leg, and thus imaging studies were requested. In MRI imaging, a mass with the maximum diameter of 3cm was found. Laboratory tests were normal, except for CRP (105 mg/dl). The patient was then referred for histological assessment. The excisional biopsy revealed a well-defined gray-pink mass measuring 3 × 2 × 2cm in the central part near the anterior margin with a distance of about 0.2cm. The microscopic study of the tumor consists of cellular nests which were separated by fibrous bands from each other. The forms of cells vary from round-shaped to fusiform with vesicular nucleus, prominent nucleolus and granular eosinophilic cytoplasm. Few multinucleated giant cells and mitosis (at most 5/10 HPF) were also seen. Some lymphocyte cells were seen in fibrous bands. Area of necrosis was also noted. In immunohistochemical (IHC) staining, antibodies that were in keeping with clear cell sarcoma were positive against the following markers:* S-100, HMB45, Melan A, and Bcl2. * Negative staining for *CK, EMA, SMA, Desmin, CD34, CD56, Myogenin, and CD57* markers was also observed (Figure 1). 

## Discussion

 Clear cell sarcoma is a slow-growing malignant tumor mainly attached to tendons in the limbs, particularly in the lower extremity. This sarcoma is slightly more common in women than in men^   7 ^. 

Clear Cell Sarcoma (CCS) of soft tissue is an infrequent and aggressive tumor. It has no signs and symptoms in the primary stage^   8 ^. CCS of soft tissue often peaks during the 20-40 range of age^   8 ^. For treatment of Clear cell sarcoma of soft tissue, the best strategy is chemotherapy in combination with radiation therapy and surgical procedures^   9 ^. Histological and immunohistochemical studies have critical role in the diagnosis of non-neoplastic, preneoplastic and neoplastic disorders^10^^,^^11^^-^^15^. 

Some important points should be considered for early diagnosis of CCS. First, this malignancy may be appeared in the limbs, particularly in the legs or thigh as a small tender spot or swelling^   9 ^. The patient has no history of trauma or any history of metabolic and inflammatory conditions. Even, family history of cancer may not be expressed by the patient^   9 ^. Second, in physical examination, a nodular soft mass might be discovered at the site of involvement that can be accompanied with gradual pain and claudication^   9 ^.

 Third, routine laboratory investigations are nonspecific ^   9 ^. As shown, positive immunohistochemical antibodies can guide the clinicians to the diagnosis of CCS. In this regard, the neoplastic spindle cell components are almost uniformly *S-100* protein positive. But, none of the antigens were specific for final diagnosis of CCS ^   16 ^. After the final diagnosis, surgical removal of the mass and any involved lymph nodes or adjacent structures followed by chemotherapy should be considered for successful treatment in most patients^14^^, ^^16^.

In 1965, Dr. Franz Enzinger attempted to clear the prominence due to his first-ever description of this unique sarcoma^   17 ^. The tumor size ranged from 0.4 cm up to 14.5 cm in dimension. Histologically, CCS displays compact nests and fascicles of uniform to minimally pleomorphic tumor cells which are delineated by dense fibrous septa. The neoplastic cells are either spindle-shaped or polygonal with abundant clear or pale eosinophilic cytoplasm and a centrally-located round to ovoid vesicular nuclei with prominent nucleoli. Mitotic activity is often low, while scattered multinucleated giant cells are seen in half of cases^   15 ^. The tumor cells are immunopositive for the common melanocytic markers, namely *HMB-45*, microphthalmia transcription factor (*MITF*), *S-100* protein, and *Melan-A* in 90%, 71%, 64% and 43% cases, respectively ^   18 ^. A reciprocal translocation t (12;22) (q13;q12) resulting in a *EWSR1/ATF1* chimeric transcript was also seen in 90% cases as a cytogenetic hallmark of CCS ^   1 ^^,^^   19 ^^,^^   20 ^.

Primary or metastatic MM, along with its histological, immunohistochemical and ultra-structural overlap constitutes the most important diagnostic mimic of coetaneous CCS^   17 ^. However, several reliable histologic yardsticks for an accurate distinction between CCS and MM has been developed by M. Hantschke^21^. CCS is mostly characterized by hyalinized sclerotic and reticulated stroma with fascicles of uniform population of tumor cells encased by delicate fibrous septa. This pattern is hardly observed in MM. Furthermore, CCS does not display any pagetoid spread of atypical melanocytes and mostly is featured tumor giant cells with characteristic wreath of multiple peripherally-placed nuclei. Ultimately, the t (12; 22) (q13; q12) translocation observed in most cases of CCS has not yet been seen in MM. Other differential diagnoses of CCS located in the extremity include paraganglioma-like dermal melanocytic tumor, clear cell myelomonocytic tumor, malignant peripheral nerve sheath tumor, and synovial sarcoma, especially the monophasic type ^   22 ^. Diagnosis is established often by a careful histological evaluation coupled with immunohistochemical demonstration of melanocytic differentiation in CCS.
